# Determinants of post-stroke depression among stroke survivors at University of Gondar Hospital, Northwest Ethiopia: a case-control study

**DOI:** 10.1186/s12883-022-02982-x

**Published:** 2022-12-01

**Authors:** Ephrem Fantu, Workagegnehu Hailu, Nebiyu Bekele, Tewodros Tsegaye, Melaku Tadesse, Mezgebu Silamsaw Asres

**Affiliations:** 1 Department of Internal Medicine, College of medicine and health sciences, University of Gondar, Gondar, Ethiopia; 2 MDR tuberculosis treatment center, University of Gondar Hospital, Gondar, Ethiopia

**Keywords:** Stroke, Post stroke depression, Determinants, Risk factors, Ethiopia

## Abstract

**Background:**

Stroke is one of the most common causes of disability among adults. Post-stroke depression (PSD) is a frequent neuropsychiatric complication in stroke patients. Despite the increasing prevalence of stroke, there is a paucity of data on PSD and its determinants among stroke survivors in developing countries like Ethiopia. We aim to assess the factors associated with PSD in survivors of stroke.

**Method:**

A hospital-based unmatched case-control study was conducted during the period of February to July 2020 at University of Gondar Hospital among stroke survivors. Study subjects were recruited consecutively. Socio-demographic and clinical data were obtained from patients’ interviews and medical record reviews. A diagnosis of PSD was made using the Patient Health Questionnaire (PHQ-9). EpiData version 3.1 was used to enter data, and SPSS version 26 was used to analyze it. Bivariate and multivariate logistic regressions were fitted to identify associated variables. The adjusted odds ratio (AOR) with 95% confidence interval (CI) and *p*-value 0.05 were used to determine the significance of the association.

**Result:**

A total of 240 stroke survivors were included in the study (80 cases and 160 controls). The mean age was 60.8 years (SD ± 14.3) with an equal sex distribution. Variables statistically associated with PSD were male gender (AOR = 3.5, 95% CI: 1.64-7.46 C, *P*-value = 0.001), subcortical location of the largest lesion (AOR = 2.42, 95% CI: 1.06–5.56, *p*-value = 0.036), severity of the stroke (AOR = 52.34, 95% CI:10.64-256.87, *p*-value = 0.000), physical disability (AOR = 5.85. 95% CI:1.94–17.65, *p*-value = 0.002), previous history of stroke or transient ischemic attack (AOR = 5.90, 95% CI:2.04–17.10, *p*-value = 0.001) and ischemic heart disease (AOR = 9.97, 95% CI:3.4-29.22, *p*-value = 0.000).

**Conclusion:**

Important factors in the occurrence of PSD in this study include prior history of stroke, physical disability, severity of the stroke, subcortical location of the lesion, male gender, and ischemic heart disease. Stroke patients with such factors need routine screening for PSD, particularly in LMICs where there is uncoordinated post-stroke care, a shortage of neurologists and mental health practitioners.

## Background

A stroke is an abrupt onset of focal or global neurological deficit as a result of a vascular cause [1]. Globally, stroke is presently the most common cause of neurological disability in adults [2]. According to WHO estimates, 15 million people worldwide suffer from strokes each year, with nearly 30% of those affected permanently disabled [3]. Early diagnosis and advances in acute stroke treatment reduced mortality. However, the prevalence of stroke has relatively remained the same and may even be on the increase [4]. The burden of stroke in developing countries is increasing, partly due to socio-demographic and lifestyle changes [5]. Community-based studies conducted in Africa revealed an age-standardized annual stroke incidence and prevalence rate of 316 per 100 000 populations and 981 per 100 000 populations, respectively [5]. In a study done at Black Lion Specialized Teaching Hospital in Ethiopia, the prevalence od stroke was found to be 19.3% [6]. 

About two-thirds of stroke survivors have residual neurological deficits, and in nearly half of them, the disability will make them dependent on others [7]. Post-stroke depression (PSD) is a common neuropsychiatric complication of stroke [8]. The Diagnostic and Statistical Manual of Mental Disorders V (DSM-V) defines PSD as a mood disorder resulting from a medical condition and characterized by an episode of major depression or a mood disorder with depressive symptoms [9].

PSD has a widely varying prevalence, ranging from 25 to 79% depending on the type of studies and diagnostic tools used [8]. However, in most parts of the world, it is under-recognized and undertreated, especially in developing countries [10].

Previous studies revealed that PSD is related to different factors, such as age, sex, ethnicity, cultural background, and education level, but the results are not always consistent [11]. The most consistent predictors of PSD so far known are physical disability, stroke severity, prior history of depression, and cognitive impairment [12].

Unlike in western countries, where PSD is extensively covered in literature, there are limited studies in African countries. Risk factors for PSD in LMICs like Ethiopia might be different due to differences in socio-economic, cultural, and quality of stroke care between the western population and developing countries. The prevalence of non-communicable diseases, including stroke, is increasing in Ethiopia. Apart from physical disabilities, neuropsychiatric morbidities like PSD are given little attention and there is a paucity of data on this. Additionally, knowing the predisposing factors for PSD will help to design strategies for prevention, early detection, and treatment of PSD in resource-limited settings like Ethiopia. The aim of this study is to assess determinant factors for post-stroke depression at University of Gondar Hospital (UoGH) in northwest Ethiopia.

## Methods

### Study design and the setting

A hospital-based unmatched case-control study was conducted during the period of February - to July 2020 at University of Gondar hospital (UoGH). The hospital is located in Northwest Ethiopia, which is 748 km from the capital, Addis Ababa. It is one of the largest hospitals in the country with a catchment population of more than 5 million people [13]. The hospital provides health services for different diseases, including care for stroke patients in a separate inpatient stroke care unit and an outpatient comprehensive neurology clinic by neurologists, trained nurses, residents, and physiotherapists.

### Study population and subjects

All stroke patients visiting the comprehensive neurology follow-up clinic or physiotherapy unit at the UoGH were the source population. Stroke patients who came for follow-up during the study period were the study subjects.

Eighteen- year-old or older patients with computed tomography (CT) or magnetic resonance imaging (MRI) confirmed stroke attacks, at least 2 weeks after the onset of the current stroke and not beyond 12 months from the onset [14] were included in the study. Stroke patients with a significant cognitive deficit (MMSE < 20), major hearing or visual difficulty, having a major psychiatric illness other than depression, those taking psychotropic agents, and aphasic patients were excluded from the study.

### Study variables

The outcome(dependent) variable was Post-Stroke Depression (PSD)and independent variables include: Socio-demographic characteristics such as age, sex, economic status, and residence; Clinical characteristics include stroke subtype, location of the lesion, severity of the stroke, time since stroke, previous history of stroke, and comorbidities such as hypertension (HTN), diabetes mellitus (DM), ischemic heart disease (IHD), and atrial fibrillation (AF); Behavioral factors, including smoking and excessive alcohol consumption.

### Sample size and sampling procedure

The sample size was calculated by taking into account the major associated factor for PSD and utilizing the two-population proportions formula in the Statistical program of the EPI INFO version 7.2.4. A 5% level of significance, 80% power, and a one-to-two case-to-control allocation ratio (1:2) were assumed in this regard. From prior studies, major physical disability following a stroke is a major risk factor for PSD, which occurs in 60% of depressed patients (cases) and 40% of patients without depression (controls) [15]. Based on these assumptions and an additional 10% for non-response, the overall sample size was 240, including 80 cases and 160 controls. A consecutive sampling method was used to recruit the 240 study subjects. Patient recruitment was done by approaching each patient during their routine scheduled follow-up visit at the neurology and physiotherapy clinic. .

### Data collection instruments and procedures

A face-to-face interview with a structured questionnaire prepared for the purpose of this study was used for data collection. The questionnaire was first prepared in English and translated into the local language (Amharic) for data collection, and then re-translated back to English for data entry and analysis while maintaining its consistency. Translation was done by two medical staff. The interview was conducted by trained data collectors consisting of interns and medical residents.

Participants were interviewed to obtain socio-demographic data and hospital medical records were reviewed to extract clinical information. .

Patient health questionnaire-9 (PHQ-9) was used to diagnose PSD [16]. It is a 9-question tool scored 0–27, and as the value increases, depression severity increases. PHQ-9 is graded as mild (0–9), moderate [10–14], moderately severe [15–19], and severe [20–27]. Clinically significant depression was considered when the PHQ-9 is 10 and above [16].

The National Institute of Health Stroke Scale (NIHSS) was used to evaluate the severity of stroke. A high NIHSS score signifies a severe stroke, and it has been shown to be the best predictor of outcome [17]. A modified Rankin scale (mRS) was used to measure the physical disability of the patients. Patients with a score 0–2 were categorized as ‘none to slight disability’ score of 3–4 as ‘moderate to moderately severe disability’ and a score of 5 as ‘severe disability/bed ridden’ [18].

### Operational definitions

Cases were those stroke survivors who fulfill patient health questionnaire-9 (PHQ-9) criteria for PSD (i.e. score > = 10) and Controls were those stroke survivors who do not fulfil PHQ-9 criteria for PSD (i.e. score 0–9) [16].

### Data processing and analysis

Data were entered into EPI-DATA version 3.1 and exported to Statistical Package for Social Sciences (SPSS) version 26 software for analysis. Patient characteristics were reported as counts (percentages) for categorical variables, and mean with standard deviation for continuous variables. Bi-variable and multi-variable logistic regression models were used to identify associated factors with post-stroke depression. Multicollinearity was checked between different variables by correlation and variance inflation factors (VIF) tests. Variables with no multicollinearity were entered to the multivariate analysis. Those variables with a *P*-value < 0.25 in the bi-variable analysis were fitted to multi-variable analysis to control for the possible effect of confounders. The crude odds ratio (COR) and adjusted odds ratio (AOR) were reported. *P*-value < 0.05 was used to declare a significant association.

## Results

A total of 476 stroke survivors were approached for inclusion in the study in order to reach the pre-determined sample size of 240 subjects. The remaining were excluded according to the exclusion criteria. Out of the 240 eligible participants, all agreed to participate in the study making a response rate of 100%.

### Socio-demographic characteristics of study participants

The mean age of study subjects was 60.8 years (SD ± 14.3). Males and females were equally represented. The majority (57.5%) of the study participants are from rural areas. More than half of the participants are unable to read and write 130 (54.17%) (Table 1).

**Table 1:** Socio-demographic and Economic characteristics of stroke patients at University of Gondar Hospital, 2021 (*n* = 240)

**Variables**	**Category**	**Cases ** ***n*** **=80**	**Controls ** ***n*** **=160**	**Total ** ***n*** **=240**
Age (years)	<40	10 (12.5%)	7 (4.38%)	17 (7.08%)
	40-59	23 (28.75%)	71 (44.38%)	94 (39.17%)
	60-79	40 (50%)	64 (40%)	104 (43.33%)
	80-99	7 (8.75%)	18 (11.25%)	25 (10.42%)
Sex	Male	51 (63.75%)	70 (43.75%)	121 (50.42%)
	Female	29 (36.25%)	70 (56.25%)	119 (49.58%)
Marital Status	Single	7 (8.75%)	13 (8.13%)	20 (8.33%)
	Married	53 (66.25%)	121 (75.63%)	174 (72.5%)
	Widowed	13 (16.25%)	14 (8.75%)	27 (11.25%)
	Separated	7 (8.75%)	12 (7.5%)	19 (7.92%)
Residence	Urban	39 (48.75%)	63 (39.37%)	102 (42.5%)
	Rural	41 (51.25%)	97 (60.62%)	138 (57.5%)
Educational Status	Unable to read and write	45 (56.25%)	85 (53.12%)	130 (54.17%)
	Primary	13 (16.25%)	41 (25.62%)	54 (22.5%)
	Secondary	7 (8.75%)	13 (8.13%)	20 (8.33%)
	Diploma/Degree	15 (18.75%)	21 (13.12%)	36 (15%)
Occupation	Civil Servant	7 (8.75%)	16 (10%)	23 (9.58%)
	Merchant	18 (22.5%)	32 (20%)	50 (20.83%)
	Farmer	22 (27.5%)	34 (21.25%)	56 (23.33%)
	Housewife	23 (28.75%)	64 (40%)	87 (36.25%)
	Retired	5 (6.25%)	2 (1.25%)	7 (2.92%)
	Unemployed	5 (6.25%)	12 (7.5%)	17 (7.08%)
Family Monthly Income	Very Sufficient	0	2 (1.25%)	2 (0.83%)
	Sufficient	13 (16.25%)	12 (7.5%)	25 (10.42%)
	Average	28 (35%)	68 (42.5%)	96 (40%)
	Insufficient	36 (45%)	64 (40%)	100 (41.67%)
	Very insufficient	0	3 (1.87%)	3 (1.25%)
	No monthly Income	3 (3.75%)	11 (6.87%)	14 (5.83%)

### Characteristics of the index stroke

More than half of the patients in the case group 49 (61.25%) have hemorrhagic stroke with subcortical location. The majority of the patients 55 (68.75%) had developed depression within the first six months after the index stroke. Among the cases, 41 (51.25%) had the lesion on the left hemisphere of the brain, and in 91 (56.87%) controls, the location of the largest lesion was in the cortex. The majority of the study participant cases 73 (91.25%) had moderate to severe stroke (NIHSS score 5–20) (Table 2).

**Table 2:** Characteristics of the index stroke in patients at University of Gondar Hospital, 2021 (*n* = 240)

**Variable**	**Category**	**Cases ** ***n*** **=80**	**Controls ** ***n*** **=160**	**Total ** ***n*** **=240**
Time since stroke	≤ 6 month	55 (68.75%)	96 (60%)	151 (62.92%)
	6-12 months	25 (31.25%)	64 (40%)	89 (37.08%)
Type of stroke	Ischemic	31 (38.75%)	105 (65.62%)	136 (56.67%)
	Hemorrhagic	49 (61.25%)	55 (34.37%)	104 (43.33%)
Size of the largest lesion	<1.5 cm	13 (16.25%)	62 (38.75%)	75 (31.25%)
	≥1.5 cm	18 (22.5%)	43 (26.87%)	61 (25.42%)
Side of the largest lesion	Left hemisphere	41 (51.25%)	69 (43.12%)	110 (45.83%)
	Right hemisphere	30 (37.5%)	57 (35.62%)	87 (36.25%)
	Both hemispheres	9 (11.25%)	25 (15.62)	34 (14.17%)
	Brain stem	0	3 (1.87%)	3 (1.25%)
	Cerebellum	0	6(3.75%)	6(2.5%)
Location of the largest lesion	Cortex	28 (35%)	91 (56.87%)	119 (49.58%)
	Sub cortex	51 (63.75%)	60 (37.5%)	111 (46.25%)
	Brain stem	1 (1.25%)	9 (5.62%)	10 (4.17%)
Severity of the stroke (NIHSS)	1-4 (minor)	6 (7.5%)	30 (18.75%)	36 (15%)
	5-20 (moderate to severe)	43 (53.75%)	124 (77.5%)	167 (69.58%)
	21-42 (severe)	31 (38.75%)	6 (3.75%)	37 (15.42%)

*NIHSS* National Institute of Health Stroke Scale

#### Clinical and behavioral characteristics

Those with depression were more likely to have a history of hypertension 54 (67.5%). All patients with previous history of stroke attack had ischemic stroke. Depressed patients were more likely to be current alcohol drinkers, but self-reported smoking did not differ between the groups (Table 3).

**Table 3:** Clinical and behavioral characteristics of stroke patients at University of Gondar Hospital, 2021 (*n* = 240)

**Variable**	**Category**	**Case ** ***n*** **=80**	**Control ** ***n*** **=160**	**Total ** ***n*** **=240**
Previous history of stroke/TIA	Yes	15 (18.75%)	5 (3.12%)	20 (8.33%)
	No	65 (81.25%)	155 (96.87%)	220 (91.67%)
History of HTN	Yes	54 (67.5%)	93 (58.12%)	147 (61.25%)
	No	26 (32.5%)	67 (41.87%)	93 (38.75%)
History of IHD	Yes	17 (21.25%)	15 (9.37%)	32 (13.33%)
	No	63 (78.75%)	145 (90.62%)	208 (86.67%)
Atrial Fibrillation	Yes	12 (15%)	27 (16.87%)	39 (16.25%)
	No	68 (85%)	133 (83.12%)	201 (83.75%)
DM	Yes	3 (3.75%)	10 (6.25%)	13 (5.42%)
	No	77 (96.25%)	150 (93.75%)	227 (94.58%)
History of Smoking	Current smoker	2 (2.5%)	1 (0.62%)	3 (1.25%)
	Previous smoker	1 (1.25%)	1 (0.62%)	2 (0.83%)
	No	77 (96.25%)	158 (98.75%)	235 (97.92%)
History of alcohol drinking	Currently drinking	11 (13.75%)	12 (7.5%)	23 (9.58%)
	Previously drinking	2 (2.5%)	11 (6.87%)	13 (5.42%)
	No History	67 (83.75%)	137 (85.62%)	204 (85%)

*TIA* Transient ischemic attack, *HTN* hypertension, *IHD* Ischemic heart disease, *DM* Diabetes mellitus

### Depression and functional status

More than two-thirds, 63 (78.75%) of the cases have moderate to moderately severe major physical disability (mRS 3–4) associated with the index stroke (Table 4).

**Table 4:** Depression and Functional status of stroke Patients in University of Gondar Hospital, 2021 (*n* = 240)

**Variable **	**Category**	**Case ** ***n*** **=80**	**Control ** ***n*** **=160**	**Total ** ***n*** **=240**
**Physical disability associated with the stroke/mRS**	0-2 (none to slight disability )	17 (21.25%)	83 (51.87%)	100 (41.67%)
	3-4 (moderate to moderately severe disability)	41 (51.25%)	65 (40.62%)	106 (44.17%)
	5 (severe disability/bed ridden)	22 (27.5%)	12 (7.5%)	34 (14.17%)
**Post stroke **	0-9 (None/mild)	0	160 (100%)	160 (66.67%)
**depression level based on PHQ9**	10-14 (Moderate)	29 (36.25%)	0	29 (12.08%)
	15-19 (moderately severe)	47 (58.75%)	0	47 (19.58%)
	20-27 (Severe)	4 (5%)	0	4 (1.67%)

*mRS* Modified Rankin scale, *PHQ9* Patient health questionnaire-9

#### Factors associated with post stroke depression

The bi-variable analysis identified that male gender, subcortical location of the largest lesion, severity of the stroke, history of ischemic heart disease, previous history of stroke or TIA, and physical disability associated with the stroke were predictors of post-stroke depression. When variables in bi-variable analysis with *P*-value < 0.25 were regressed in multi-variable analysis, male gender (AOR = 3.5, 95% CI: 1.64-7.46 C, *P*-value = 0.001), subcortical location of the largest lesion (AOR = 2.42, 95% CI: 1.06–5.56, *p*-value = 0.036), severity of the stroke (AOR = 52.34, 95% CI:10.64-256.87, *p*-value = 0.000), physical disability (AOR = 5.85. 95% CI:1.94–17.65, *p*-value = 0.002), previous history of stroke or TIA (AOR = 5.90, 95% CI:2.04–17.10, *p*-value = 0.001) and ischemic heart disease (AOR = 9.97, 95% CI:3.4-29.22, *p*-value = 0.000) were found to be predictors of PSD (Table 5).

**Table 5:** Bi-variable and multivariable logistic regression analysis of factors associated with PSD in stroke patients at University of Gondar Hospital, 2021 (*n* = 240)

**Variables**		**COR(95% CI)**	***P*** **-value**	**AOR (95%CI)**	***P*** **-value**
Sex	Male	2.261 (1.301-3.929)	0.004	3.497 (1.639-7.461)^a^	.001
	Female	1			
Type of stroke	Ischemic	0.331 (0.190-0.578)	0.000	0.340 (.150-.769)^a^	0.010
	Hemorrhagic	1			
Location of the largest lesion	Cortex	1		1	
	Sub cortex	2.762 (1.571-4.858)	0.000	2.425 (1.06-5.556)^a^	0.036
	Brain stem	0.361 (0.044-2.975)	0.344		
Severity of the stroke (NIHSS)	1-4 (minor)	1		1	
	5-20 (moderate to severe)	1.734 (0.675-4.450)	0.253	2.794 (.900-8.679)	.076
	21-42 (severe)	25.833 (7.491-89.085)	0.000	52.34 (10.64-256.87)^a^	.000
History of IHD	Yes	2.608 (1.226-5.548)	0.013	9.967 (3.399-29.224)^a^	.000
	No	1		1	
Previous history of stroke/TIA	Yes	6.77 (2.956-15.505)	0.000	5.902 (2.036-17.103)^a^	0.001
	No	1		1	
Physical disability associated with the stroke/mRS	0-2 (none to slight disability )	1		1	
	3-4 (moderate to moderately severe disability)	3.080 (1.605-5.911)	0.001	4.118 (1.818-9.329)^a^	.001
	5 (severe disability/bed ridden)	8.951 (3.728-21.489)	0.000	5.850 (1.939-17.650)^a^	.002

^a^Statistically significant

## Discussion

We found that the risk of depression after stroke was significantly associated with male gender, location of the largest lesion, ischemic heart disease, severity of the stroke, major physical disability, and prior history of stroke or TIA.

The association of male gender with PSD is consistent with previous studies from other countries such as India and Sweden [19, 20]. The location of the lesion was one of a well-established clinical risk factors for development of PSD. In a recent study from China, basal ganglia lesions and lesions located in the left hemisphere regions have been associated with the development of PSD [21]. In our study, PSD was more frequently observed in patients with subcortical lesions compared to cortical lesions (63.75% vs. 35%, *P* = 0.036). This finding is also consistent with a recent study from India and the DEPRESS study from France [22, 23].

In this study, the severity of the current stroke was found to be an important risk factor for PSD. This is consistent with findings from a recent Nigerian study involving 112 stroke patients [24]. Other studies from Finland and the USA also reported similar findings [25, 26].

Having a concomitant diagnosis of ischemic heart disease (IHD) diagnosed by review of ECG and/or echocardiography was significantly associated with an increased likelihood of developing PSD (AOR = 9.967(3.399–29.224)^)^. The correlation between PSD and vascular risk factors is less clear. A single study from Netherlands which recruited most patient in the first week from stroke onset, revealed angina pectoris as an independent predictor of PSD [27]. However, established history of stable IHD has not been reported to be an independent predictor for PSD Cardiac diseases, including IHD may contribute to physical disability in addition to stroke-related disability, and by doing so, may increase the risk of PSD.

Consistent with previous studies, this study showed that history of previous stroke or TIA is associated with PSD [22, 28].

Stroke survivors often suffer some degree of long-term impairment, such as partial or complete loss of locomotion. Other possible areas of impairments include activities of daily living, cognition, and communication skills [29]. Our study also revealed that a significant number of stroke survivors with physical disability as a result of the index stroke have developed PSD. This finding is in agreement with a recent report from Egypt [30]. Several other studies from Canada, the UK, France, and the USA have reported similar findings [25, 26, 31, 32].

Data on predictors of stroke related depression is scanty in Low-and-Middle Income Countries (LMICs). Our study is one of the fewest studies in LMICs, particularly in Sub-Saharan region to assess predictors PSD. Most of the predictors of PSD in our study were reported in previous studies. This could be due to the growing burden of non-communicable diseases, urbanization and aging of the population that has resulted in shifting the diseases epidemiology from communicable to non-communicable diseases’. The prescience of such several predictors of PSD may indicate a greater burden of undiagnosed and untreated depression among stroke survivors in the region where there is access to quality stroke care.

Prior studies reported that the duration of the stroke was mentioned as a risk factor for PSD with a high prevalence in the first year [33]. Additionally, the age of the patient, place of residence, family income, and educational status were not found to be predictors of PSD in this study. This contradicts the findings of some previous studies [34, 35]. These differences could be due to the population characteristics. As opposed to prior studies from developed nations, our study population were relatively younger age having poor educational status and majority being from rural areas.

### Strengths and limitations of the study

Our study has a good sample size and utilized a case-control design study method, which is most appropriate to identify factors associated with PSD. However, the findings of this study should be interpreted within the context of the following few limitations. Stroke subjects had never been screened for depression before onset of stroke as part of routine care. As this is a cross-sectional study, it was not possible to distinguish between pre-existing depression and PSD. However none of them were taking antidepressant medication. Highly disabled stroke participants, such as those with aphasia and deafness, who usually account for higher rates of PSD, were excluded from the study due to the lack of standardized diagnostic tools in such patients. This study used self-report questionnaires with rank-order scales to determine perceived depression using the interview method. Possible response bias may have been introduced into the results as patients may give socially desired responses so as not to lose face.

## Conclusion

Post-stroke depression is a common sequel of stroke. There are many factors that have effects on PSD. Prior history of stroke, physical disability, and severity of stroke, subcortical location of the lesion, male gender, and ischemic heart disease are important factors in the occurrence of PSD in the current study. Stroke patients with such factors need routine screening for PSD, particularly in LMICs where there is uncoordinated post-stroke care, a shortage of neurologists and mental health practitioners.

## Data Availability

The datasets used and/or analyzed during the current study are available from the corresponding author on reasonable request (email: workhailu@yahoo.com).

## Abbreviations

AF: Atrial Fibrillation
AOR: Adjusted Odds Ratio
CI: Confidence Interval
COR: Crude Odds Ratio
CT: Computed Tomography
DM: Diabetes Mellitus
DSM-V: Diagnostic and Statistical Manual of Mental Disorders-V
HTN: Hypertension
IHD: Ischemic Heart Disease
IRB: Institutional Review Board
MRI: Magnetic Resonance Imaging
MRS: Modified Rankin Scale
NIHSS: National Institute of Health Stroke Scale
PHQ-9: Patient Health Questionnaire-9
PSD: Post-Stroke Depression
SD: Standard Deviation
SPSS: Statistical Package for Social Sciences
TIA: Transient Ischemic Attack
UoGH: University of Gondar Hospital
USA: United States of America
WHO: World Health Organization
